# First Mile program: North-South partnership experience with health system strengthening in Mbarara, Uganda

**DOI:** 10.1080/16549716.2025.2607907

**Published:** 2026-01-15

**Authors:** Peter K. Olds, Stephen Asiimwe, Annet Kembabazi, Andrew Ainomugisha, Jodi Ansel, Joseph Ngonzi, Grace Nambozi, Edgar Mugema Mulogo, Moses Ntaro, Data Santorino, Raymond Atwine, Johnes Obunguloch, Rose Muhindo, Grace Kansiime, Harriet Nabulo, Jane Kasozi Namagga, Mary Sebert, Peter Chris Kawungezi, Brian Turigye, Dennis Nelson Wandera, Nuriat Nambogo, Abraham Birungi, Louise C. Ivers, Celestino Obua

**Affiliations:** a Center for Global Health, Massachusetts General Hospital, Boston, MA, USA; b Department of Medicine, Harvard Medical School, Boston, MA, USA; c Faculty of Medicine, Mbarara University of Science and Technology (MUST), Mbarara, Uganda; d Global Health Collaborative, MUST, Mbarara, Uganda; e CAMTech Uganda, Mbarara, Uganda

**Keywords:** collaborations, capacity building, equity, clinical care delivery, clinical education, research and innovation support

## Abstract

Uganda’s public healthcare system faces significant systemic challenges in delivering care, contributing to poor health outcomes. In response, a North-South partnership between Massachusetts General Hospital (MGH), Mbarara University of Science and Technology (MUST), and Mbarara Regional Referral Hospital (MRRH) implemented the First Mile Project, a seven-year initiative (2018–2024) designed to strengthen the regional health system in southwestern Uganda. Guided by local priorities, the program aimed to improve access to care, enhance clinical capacity, expand medical education, and promote research and innovation. Project activities were devised collaboratively between investigators at MGH and MUST/MRRH, with robust local leadership and oversight. Key initiatives included clinical staffing in critical departments, construction of a regional isolation ward and oxygen plant. Additionally, the program provided scholarships for medical training, support for community-based care and continuing medical education, and provision of research and innovation grants. The program trained over 1500 health workers, supported 93 scholarships, increased access to specialized clinical services, and facilitated community outreach to thousands of households. The project also awarded 74 research grants and supported over 30 locally incubated innovations. Through this comprehensive, equity-focused approach, First Mile demonstrated how collaborative, locally driven partnerships can effectively strengthen health systems in low-resource settings. Lessons from the initiative underscore the importance of sustained local leadership and integrated clinical and research efforts. A 20-year history of collaboration and mutual trust ensured open dialogue between partners and helped support the success of the project. The First Mile model provides a promising example for future health system strengthening initiatives globally.

## PAPER CONTEXT

- **Main findings:** Driven by local priority-setting, a North-South partnership significantly strengthened healthcare delivery, education, and innovation capacity in southwestern Uganda through a seven-year coordinated health systems strengthening initiative.
- **Added knowledge**: This work demonstrates that through equitable priority setting and integrating clinical care, education, and research within a health system strengthening framework, North-South partnerships can achieve broad, sustainable improvements in care delivery in low-resource settings.
- **Global health impact for policy and action**: The First Mile model offers a replicable, equity-focused blueprint for North-South partnerships to design future health system investments that center on local ownership, long-term partnership, and integrated service delivery.

## Background

Uganda’s public healthcare system faces significant challenges that impede the delivery of effective and equitable healthcare for the country’s population of 48 million [1]. Uganda lags behind the world for life expectancy at 66 years, with major causes of death from preventable causes, like malaria, HIV/AIDS, and tuberculosis (TB) [2]. Major challenges to care delivery stem from systemic underfunding and patient poverty, and include shortages in adequately trained health professionals, infrastructure deficiencies, and supply chain issues [3–5]. Properly addressing these barriers to adequate healthcare delivery in Uganda is critical to improving health outcomes in the country.

As part of the Sustainable Development Goals (SGDs), there is a call among the global and Ugandan medical communities for health system strengthening to broadly improve access to safe, effective, affordable health services for all [6–8]. Additionally, the World Health Organization’s (WHO) goal for Universal Health Coverage (UHC) by 2030 stresses the importance of work improving general access to care, rather than focused on a single disease or population [9]. Finding programs that can effectively bolster the healthcare workforce and improve healthcare delivery across numerous disciplines is critical to achieving UHC and meeting the SDGs in Uganda.

North-South partnerships, collaborative efforts between countries or institutions in the Global North (developed countries) and the Global South (developing countries), have been promoted as an effective means of capacity building in low- and middle-income countries (LMICs) through shared knowledge, expertise, technology, and financial resources [10]. Effective North-South partnerships are increasingly recognized as those that prioritize equity, mutual benefit, and local ownership, and have been shown to be successful in improving health research and educational capacity in LMICs [11,12]. While many North-South partnerships focus on research and educational capacity, there is a growing need for partnerships to focus on improving general access to care to address health disparities and meet SDGs.

With this growing need to address health system issues in Uganda, our partnership, a collaboration between Massachusetts General Hospital (MGH) in Boston, MA, Mbarara University of Science & Technology (MUST), and Mbarara Regional Referral Hospital (MRRH) in Mbarara, Uganda, implemented a 7-year health system strengthening project in Mbarara, Uganda, entitled the *First Mile Project: Powering the Academic Medical Center to Deliver Healthcare in the Community in Uganda* (First Mile). First Mile was developed as a program with priorities set by clinicians and researchers at MUST and its teaching hospital, MRRH. Through a mix of educational scholarships, health workforce training and salary support, research and innovation advancement, and the development of innovative clinical delivery programs, First Mile aimed to improve access to a broad range of health services in the southwestern region of Uganda. This paper discusses the program, presents its results, and explores how such a program, implemented through a North-South partnership, can be improved upon in the future.

## Program overview

### Location

The First Mile project was implemented in the southwestern region of Uganda, with a population of approximately 6–8 million and whose capital is Mbarara city, situated 250 km southwest of Kampala. Uganda has a Gross Domestic Product (GDP) per capita of 1014USD with 6.5% of GDP going towards healthcare, equating to $43 per capita [2]. Roughly 42% of the population lives below the international poverty line, with catastrophic health bills driving 4% of the population into poverty each year [13,14]. There is a severe shortage of trained clinicians, with one registered nurse for every 11,000 people and one physician for every 25,000 people [14]. Health facilities face significant difficulties in adequately caring for patients due to lack of training, supplies, and facility management [15,16].

### The Global Health Collaborative (GHC) at MUST

The Global Health Collaborative is an MGH-MUST-MRRH partnership between Massachusetts General Hospital (MGH), Mbarara University of Science and Technology (MUST), and Mbarara Regional Referral Hospital (MRRH) in Uganda spanning over two decades. The resources and strengths of each institution are used to address critical health challenges in Uganda through research, medical education, and community initiatives. The GHC works to foster scientific and development growth and leadership in Mbarara and is led by and comprised of Ugandans. A resource to the entire MUST research and development community, the GHC promotes cooperation, efficiency, and accountability, by sharing expertise, facilities, equipment, information, and support among all projects under their collective banner. This includes IRB support, IT assistance, help with the Community Advisory Board, community fora for technical and program exchanges, community events, workspace and related support, and visitor assistance.

**Figure 1.**
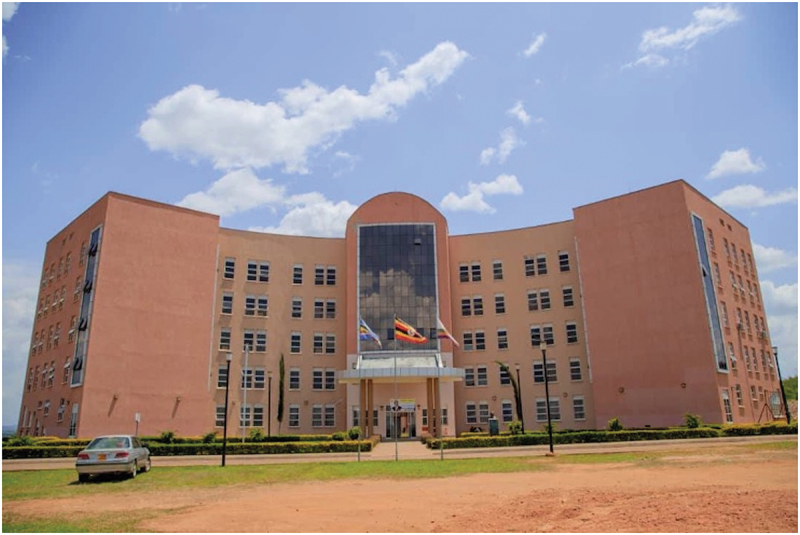
Photo of Mbarara University of Science and Technology.

**Figure 2.**
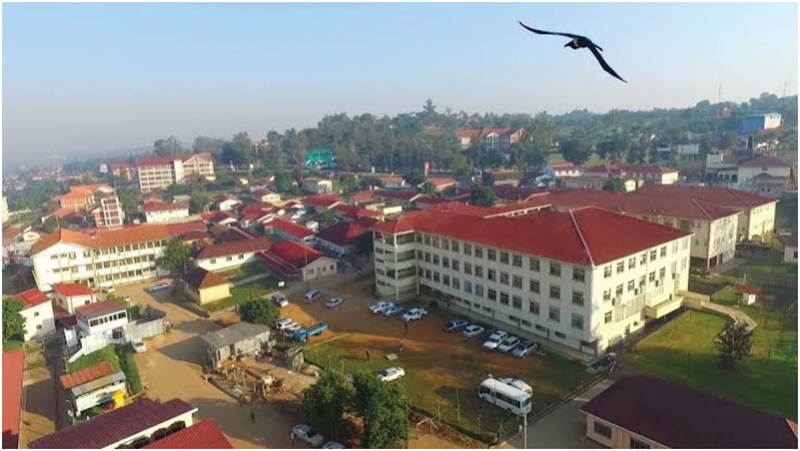
Photo of Mbarara Regional Referral Hospital.

MUST is a public university established in 1989 with a focus on science and technology and community engagement (Figure 1). The university has 6 faculties with approximately 6000 undergraduate students and 1300 postgraduate students. MRRH is a 600-bed teaching hospital affiliated with MUST that serves the southwestern region of Uganda with a catchment area of 6–8 million people offering both medical and surgical subspecialty services (Figure 2). Medical services are provided free of charge by policy, although frequent stockouts of medications and sundries require individuals to pay for much of their care out of pocket since there is no universal health insurance.

Established in 2004, the partnership is governed through the Director of the MGH Center for Global Health, as well as the Vice Chancellor at MUST and Hospital Director at MRRH. Memoranda of Understanding are updated regularly to reflect the ongoing commitments between the institutions and shared priorities. The Director of the GHC in Mbarara and the Assistant Director for Uganda Programs at the MGH Center for Global Health work together to manage and oversee daily operations of the collaboration. The partnership has prioritized research and clinical training as well as continuous mentoring. The partnership supported the development of MUST’s Master’s in Nursing program, an advanced program training nursing specialists in critical care, maternal health, pediatrics, and mental health. The partnership has fostered innovation by supporting the Consortium for Affordable Medical Technologies (CAMTech), which incubates and nurtures locally developed solutions to local health challenges. Intellectual Property is shared by MUST and the Ugandan teams. The partnership also was a catalyst in the establishment of the MUST Grants Office (MGO) in 2010 [17]. Since its formation, the MGO has grown substantially and allows Ugandan researchers to independently apply for and manage research and clinical projects from outside entities. In 2024, MGO managed 48 active awards from both Ugandan and international sponsors [18].

## Program description

The First Mile Program was launched in 2018 with the goal of improving patient care in the southwestern region of Uganda through academic, nursing and community-based initiatives. Its primary objectives were to 1) Empower nursing leadership to develop and implement innovative models of care; 2) Establish MUST as a premier academic medical center focused on community-based healthcare delivery, research and innovation for East Africa; 3) Equip the MRRH and affiliated community health facilities to better understand and meet the burden of disease; and 4) Leverage technology innovation and co-creation to support patients, community health workers, and nurses (Table 1).

**Table 1.** First Mile program overview.

Program Goals	Initial Period (2018-2020)	COVID-19 Period (2020-2021)	Final Period (2022-2024)
1. Empower nursing to implement innovative models of care	● Community Nursing Program: ○ 1 nurse placed in 1 school● MUST nursing student community placements● Leadership training and research dissemination at MGH		● Community Nursing Program:○ 3 nurses placed in 3 schools● MUST nursing student community placements● Leadership training at MGH
2. Equip MRRH to meet the burden of disease	● Salary and materials support:○ Oncology○ Intensive Care Unit	● Constructed a 44-bed isolation unit with new oxygen plant.● Salary support:○ Oncology, isolation unit● Feeding program support	● Salary support: ○ Oncology, isolation unit, emergency department, medical ICU, dialysis, high-risk obstetrics, neurosurgical ICU, biomedical engineering● Materials support:○ Biomedical engineering, pathology● Feeding program support
3. Establish MUST as a premier academic center focused on community-based care	● VHT program:○ Training, mobilization, medication support● MUST undergraduate student community health project support	● Provided training to peripheral facilities on isolation, PPE, infectious control, and surveillance.	● Post-graduate student community placements● Continuing Medical Education courses● Clinical Attachments at MRRH
4. Leverage local innovation and research.	● Salary and materials support for CAMTech:○ Ran 2 regional Hackathons○ Seed funding for 6 teams		● Salary and materials support for CAMTech:○ Ran 3 regional Hackathons○ Seed funding for 9 teams● 74 research grants awarded to early-stage researchers
Scholarships	Master’s in Medicine and Master’s in Nursing scholarships to MUST		

MUST:Mbarara University of Science and Technology; MRRH: Mbarara Regional Referral Hospital; MGH: Massachusetts General Hospital; ICU: Intensive Care Unit; VHT: village health team; PPE: personal protective equipment; CAMTech: Consortium for Affordable Medical Technologies.

An overview of the First Mile program, highlighting the 4 primary goals of the program and broken down by the three periods of the project.

Program goals and all final decisions on activities were set in a collaborative fashion between the Director of the MGH Center for Global Health, Vice Chancellor at MUST, and Hospital Director at MRRH. Program activities were developed in collaborative discussions between clinicians in respective departments. For example, nursing leadership at MUST and MRRH worked with the Director of Nursing at the MGH Center for Global Health to develop activities for Objective 1. This process was aided by historical research, clinical, and educational activities between these departments and ensured First Mile addressed local problems with local solutions. Program activities, as well as conflict resolution and manuscript authorship, were managed by the Director of the GHC and the Assistant Director for Uganda Programs at the MGH Center for Global Health.

The project had three time-periods, with an initial period from 2018 to early 2020, followed by a bridge period of limited implementation due to funding interruption at the time of renewal that aligned with the COVID-19 pandemic, and then resumption of full program activities from 2022 through end 2024.

During the initial period, the program supported a variety of activities across the university and hospital. In the Nursing department, the program supported leadership training, and a community-based nursing program, which placed a nurse in a local primary school and supported undergraduate training in community nursing. Clinically, the program supported a hospital feeding program for malnourished and cancer patients, as well as provided support for a physician, nurse, and pharmacist in the pediatric and adult cancer wards at MRRH. The program also supported Village Health Teams (VHTs) in Bugoye, Uganda. VHTs are community-selected lay volunteers who are trained to promote health awareness and provide very basic medical services. In addition to the training the Ministry of Health provides VHTs, through First Mile, we specifically trained VHTs to provide pediatric malaria treatment and prenatal support to pregnant women. Additionally, the project supported start-up innovations at CAMTech with seed funding and provided scholarships for students in Master’s in both Medicine and Nursing at MUST.

**Figure 3.**
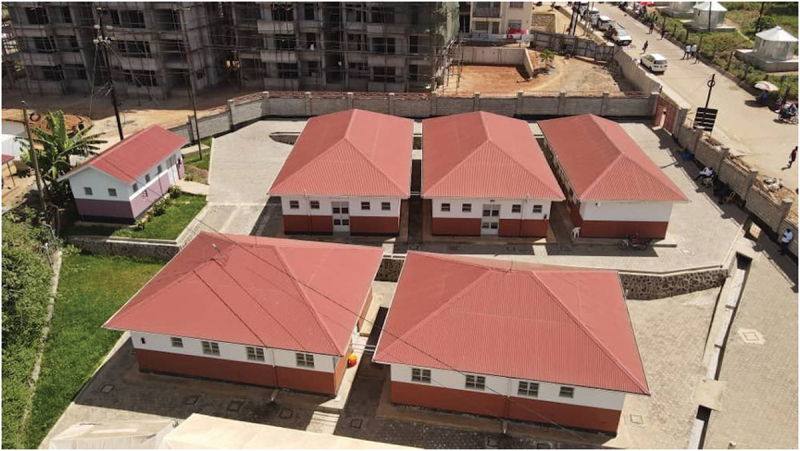
Photo of the isolation unit built at Mbarara Regional Referral Hospital through the support of First Mile.

In early 2020 at the beginning of the COVID-19 pandemic, Uganda instituted country-wide socio-geographical movement restrictions where transportation and social services were severely limited, and businesses and institutions were temporarily closed. In response to the threat of the pandemic to southwestern Uganda and the societal ‘lockdowns,’ the teams at MGH, MUST, and MRRH recognized the urgent need to redirect resources to respond to the pandemic. Given the lack of appropriate facilities to care for patients with COVID-19, the program designed, constructed and equipped a 44-bed isolation ward at MRRH, staffed it with trained personnel, and installed a new oxygen plant to meet patients’ critical oxygen needs (Figure 3). The program also supported a COVID-19 vaccination team. Since its development, this unit has been further adapted to be the special pathogens unit/infectious disease unit of both the hospital and region, handling highly infectious diseases of epidemic or pandemic potential like viral hemorrhagic diseases, anthrax, Mpox, among others.

The First Mile program funding was renewed, and phase 2 began in early 2022. The program’s goals remained the same, though review provided an opportunity for adjustments to enhance program effectiveness and address new challenges in the region. Additionally, prior to the pandemic, individual clinical groups ran parts of First Mile without clear central leadership, leading to inefficiencies with management and Monitoring and Evaluation, prompting the designation of a full-time Ugandan director in 2022. Modifications to program activities were made in shared decision-making meetings between Ugandan and MGH teams.

In the nursing department, the community-based nursing program expanded to have community nurses placed in 2 additional primary schools. Clinical work at MRRH increased to now support Oncology, Pathology, Emergency, Highly Infectious Disease (isolation ward clinical team), Intensive Care, High-Risk Obstetrics, Dialysis, Neurosurgery, and Biomedical departments. Support was predominantly salaries, though it also included support for critical materials (i.e. reagents for pathology, repair materials for biomedical engineering). Given the project had met its targets for VHT support and due to changes in local priorities, community work shifted from supporting VHTs to developing innovative educational programs for health providers at 50 health facilities in the southwestern region. These educational programs were 1) formal Continuing Medical Education (CME) courses; 2) Two-week community rotations for post-graduate medical trainees; and 3) clinical attachments for primary care providers at MRRH for focused and specialized training. The program also supported research capacity, providing 74 research grants awarded to early-stage Ugandan researchers, in addition to research training and mentorship to awardees. Finally, First Mile provided continued support to CAMTech, and funded Master’s scholarships at MUST.

## Program results

### Education

Over the course of the program, the First Mile program trained over 1500 health providers across the region, through scholarships, research support, and CME (Table 2). At the community level, First Mile aimed to provide ongoing education to support primary care providers. This led to the support and training of 110 VHTs on areas of malaria treatment and prevention and prenatal care. These providers ultimately were able to improve the health practices of 3800 households living in 22 communities by increasing knowledge and skills related to infection prevention and hygiene. In broadening this community work in the second half of the project, First Mile moved to provide CME programs to 80 health providers across the southwestern region. Topics included emergency medicine, anti-microbial resistance, evidence-based planning, and diagnosis and management of non-communicable diseases.

**Table 2.** Summary of First Mile educational programs.

Program Type	Outputs	Focus Area/Details
Master’s degree scholarships at MUST	93 individual scholarships awarded	**Nursing**: Critical Care, Pediatrics, Psychiatry, Midwifery. **Medicine**: Internal Medicine, OBGYN, psychiatry, anesthesia, ENT, ophthalmology, surgery, radiology, pathology.
MUST medicine post-graduate community placements	164 students placed in 50 rural facilities	**Departments**: Anesthesia, ENT, Emergency Medicine, Laboratory Science, Ophthalmology, OGBYN, Surgery, Biomedical engineering, Community Health. **Services revitalized**: Surgical operations, eye clinics, ENT clinics, blood transfusion services, OBGYN clinics, diabetes and hypertension clinics, biomedical engineering
Continuing Medical Education	4 CME courses; 80 providers trained from 50 facilities	**Topics**: Emergency medicine, Anti-microbial resistance, Evidence based planning, Diagnosis and management of non-communicable diseases.
Community Nursing Program	3 schools and communities; 547 MUST nursing students trained	**School placements**: 3 schools were identified by level of need. Nurses were placed at each school to provide nursing care to students and health education to the community. **Community nursing education**: A total of 547 MUST nursing students were placed in these 3 communities, where they performed 577 home visits and 562 health education talks.
Clinical Attachments	31 primary health care providers trained	**Departments**: Anesthesia, ENT, Pediatrics, Ophthalmology, Laboratory Science, OBGYN, Dermatology
Village Health Teams	110 total teams supported	**Focus**: Trained and supported VHTs in malaria and pre-natal care. **Impact**: Grew the program from 8 to 22 villages, impacting 3800 households. 4500 under 5 children were evaluated for malnutrition and malaria.
Nursing leadership	8 MUST/MRRH faculty trained at MGH	**Topics**: Reflective practice, communication skills, feedback and feed forward, team building, conflict management, advocacy and lobbying, emotional intelligence, problem solving, managing up and across, supervision. **Sustainability**: 2 ‘trainers of trainees’ were trained, who have subsequently trained 24 nurse leaders at MRRH.

MUST:Mbarara University of Science and Technology; MRRH: Mbarara Regional Referral Hospital; MGH: Massachusetts General Hospital; ENT: Ear Nose and Throat; CME: continuing medical education; OBGYN: obstetrics and gynecology; VHT: village health team.

A summary of the educational programs of First Mile, outlining measured outputs with accompanying details of the programs.

With some facilities requesting more specialized training to improve care for their patients, our team developed a clinical attachments program, bringing 31 providers from peripheral centers to the regional referral hospital for hands-on training in a broad range of specialties (Table 1). We also supported community rotations for post-graduate physicians, a governmental program that had largely gone unfunded. We placed 164 MUST postgraduates across 25 health facilities from a range of departments (Anesthesia, Ear Nose and Throat, Emergency Medicine, Laboratory Science, Ophthalmology, OGBYN, Surgery, Biomedical engineering, Community Health), and these placements allowed the establishment or revitalization of critical clinical services (Table 2).

At the university level, the program provided scholarships to 93 students pursuing Masters degrees in medicine and nursing at MUST. These scholarships from First Mile have since been shown to have increased access to general and specialty services across Uganda [19,20]. Additionally, the program developed a leadership course that brought 8 nurse educators from MUST to MGH in Boston, MA for lectures and clinical shadowing. To promote sustainability, the final training was focused on ‘Training the Trainer’ to develop a cadre of leadership trainers in Mbarara. These trainers have subsequently trained 24 nurse leaders at MRRH. Additionally, our nursing leadership led 5 trauma courses, four held in Uganda and one in Rwanda which was co-facilitated by two Ugandans from prior trainings.

Finally, the Community Nursing program brought a focus of community nursing to nursing students at MUST. Through 577 home visits, 562 health education talks to students and the community, as well as placement of 3 nurses at primary schools in the Mbarara area, the First Mile team provided community nursing education for 547 MUST nursing students.

### Clinical delivery

In response to hospital-identified needs and priorities, the First Mile program provided multidisciplinary support across MRRH, ultimately paying salaries for 30 health professionals and providing technical assistance across 9 departments, namely oncology, emergency, high-risk obstetrics, intensive care, dialysis, neurosurgical, and biomedical engineering departments.

As noted above, First Mile co-designed and supported the construction of an isolation ward with an accompanying oxygen plant in response to the COVID-19 pandemic, the only unit of its kind in the region. It has also provided safe testing sites for suspected cases of Ebola and Marburg virus infections. During the First Mile program, the team that staffs the ward vaccinated 14,537 individuals against COVID-19 and trained over 400 healthcare providers in how to identify and test for hemorrhagic fevers.

In the Cancer Unit, which provides care for both adult and pediatric cancers, First Mile provided technical and clinical support and assumed salary support for 11 nurses, a social worker and pharmacist. This team oversaw a significant scaling up of cancer treatment at MRRH. Due to increased detection and increased retention in care, our team oversaw an 187% increase in cancer patients actively enrolled at MRRH between 2018 and 2024. By end of the program, the pharmacist was preparing approximately 15,000 chemotherapy infusions per year, and the team cared for 993 new patients in 2024 with over 6000 patients in active care.

**Figure 4.**
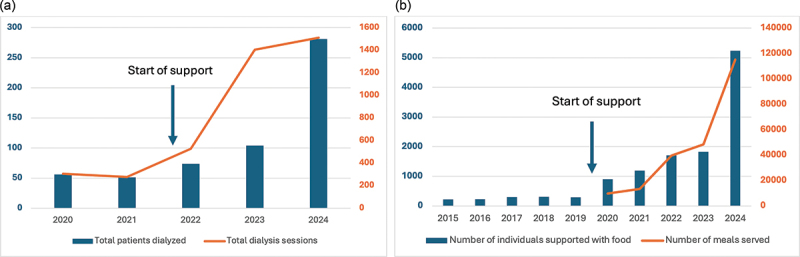
Outcomes from First Mile’s support of the MRRH dialysis unit and nutrition program. (a) Demonstrates total patients dialyzed and total dialysis sessions conducted between 2020 and 2024, identifying that First Mile support for the dialysis unit began in 2022. (b) Demonstrates total individuals fed and total meals served by the MRRH nutrition program between 2015 and 2024, identifying that First Mile support for the nutrition program began in 2020.

In the High-Risk Obstetrics unit, where First Mile supported 80% of the staff, there has been a significant decline in in-hospital mortality from 3% mortality among mothers admitted to the ward in 2022 to below 1% in 2024. In the pathology department, there has been a 65% reduction in biopsy result turnaround time since First Mile started support in 2018. Pathology turnaround time improved from 28 days in 2018 to an average of 10 days in 2024, despite a 360% increase in cases over that same time. In the dialysis department, First Mile salary support contributed to the 500% increase in dialysis sessions enhancing the government’s lowered dialysis costs for patients (Figure 4). By the end of the program, the dialysis unit at MRRH was the third largest public dialysis unit in the country.

First Mile also supported a feeding program that provided free meals to children with malnutrition and cancer as well as their caregivers, patients with tuberculosis, and other high-risk patients in the hospital. The feeding program eased the financial burden of staying at the hospital for our most disenfranchised patients, allowing them to continue with critical treatment. Since 2020, there was a five-fold increase in total meals provided and we doubled the number of individuals fed (Figure 4).

### Research and technology

First Mile expanded and strengthened a strong community of research and technology through research grant funding to Ugandan researchers and by supporting medical innovations incubated at CAMTech. Between 2018 and 2023, First Mile awarded 74 research grants worth a total of 132,290 USD. These grants went to support early career researchers with a focus on multidisciplinary work and improved care delivery in the region.

**Figure 5.**
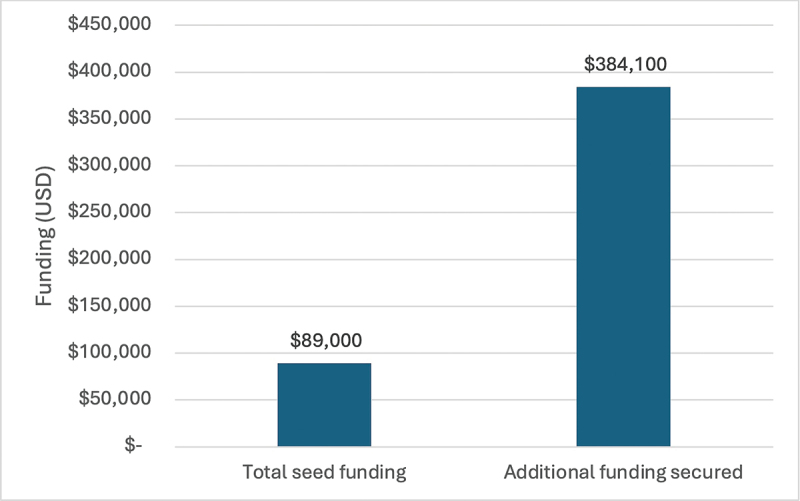
CAMTech seed funding impact. Funding provided by First Mile for CAMTech projects and the additional funding in us dollars (USD) the teams were able to secure from outside awards and funders.

CAMTech grew significantly under First Mile with the total number of projects incubated at CAMTech increasing from 15 in 2018 to 32 in 2024. First Mile also supported 5 Hackathons that attracted innovators from around East Africa for collaborative ideation and problem solving around local health challenges [21]. From the Hackathon, the top three teams were given seed funding awards, with a total of 12 seed awards provided during the First Mile program. A total of 89,000 USD was awarded the the 12 innovator teams to support prototype development, intellectual property filing, and research for proof of concept, among others. After successful completion of these milestones from the seed grants, the funded teams were subsequently able to secure an additional additional 384,100 USD in total from other funding institutions (Figure 5). CAMTech teams won several innovation awards and recognitions around the African continent and beyond, and CAMTech is poised as a leader in developing medical innovations on the continent.

### Program innovations

We used a mix of local expertise and systems thinking to bring innovative strategies to service delivery in First Mile [22,23]. In one example, our teams realized that traditional CME wouldn’t be sufficient in improving care in peripheral facilities nor would such courses fully meet the needs of the providers. This led to the development of the clinical attachments program that focused on targeted, hands-on learning in a ‘train the trainer’ methodology to further propagate this knowledge. In a second example, our team noted the importance of community nursing programs to better connect the academic medical center with communities in the region. Ultimately, the community nursing program increased community engagement through embedded nurses in local communities but also served as a training site for undergraduate nurses. In a third example, rather than just focusing on service delivery, we followed the WHO’s health system building blocks in providing support for leadership and governance systems through CME trainings and community health engagements [16,24]. While often overlooked in health system strengthening, we found that supporting leadership and governance at our health facilities not only helped implement First Mile activities, but our stakeholders reported that it broadly improved facility operations.

## Discussion

In this paper we described the First Mile Program, which was a local priority-led health system strengthening initiative meant to bolster the healthcare system in the southwestern region of Uganda. The project spanned seven years from 2018 through 2024, supporting an array of interconnected activities related to education, clinical support, as well as research and innovation. The program was wide-ranging in its activities but ultimately showed significant promise in improving care delivery and supporting innovations in the region. In describing a program of this scope, we hope to highlight in this discussion what we learned from the project and how future collaborative health system strengthening projects can be more sustainable. In so doing, we hope that this will help future teams around the world build better programs to improve healthcare in impoverished settings.

Firstly, this project highlighted the benefits of having priorities set and programs developed by local experts. This is in line with efforts within the global health community to transition from priorities led by actors in the global north towards support for capacity development in the global south and more equity in priority setting and resource allocation [25]. We found this approach led to creative solutions to health problems, especially true in our community health-based educational programs and the community nursing program.

During First Mile, there were several instances where local teams were unhappy with decisions that may have been seen as driven by donor priorities or Boston-based priorities. As mentioned, final First Mile programmatic decisions involved both Boston and Ugandan-based teams which helped increase transparency and eased conflict resolution given that decisions were not unilateral in nature. While our approach was facilitated by a history of collaboration and trust developed over the course of our 20-year collaboration, having clear processes for program decisions likely can help increase trust in younger collaborations.

The idea of locally driven priorities is not new, and is part of a growing movement for decolonizing global health and pushing for equitable opportunities at policy-setting, funding, and publication, to name a few [26]. Additionally, having locally driven priorities is a first step towards aid localization, where control, decision-making, and resources for health system strengthening projects are provided to local actors and communities [27]. We feel that opening access to resources for the talented clinicians in Uganda and other impoverished countries will allow global health work to grow exponentially, addressing locally important challenges with culturally appropriate solutions. In pushing for projects to be led by local stakeholders, prior work has highlighted the need for sufficient groundwork and support be in place to help ensure success [28].

We have found that partnerships must work together to envision what a sustainable future looks like for their health system strengthening projects and collaboration. Identifying limitations in local capacity must happen early in a project to support transparency and ensure project activities are working towards improving those limitations. One example of a limitation our partnership encountered was the need for MUST to effectively manage their research grants. This led to the development of the MGO in Mbarara, Uganda [11,17,29]. As noted earlier, the development of the MGO predated the First Mile grant and took significant time, collaboration, and effort but is now able to effectively manage the First Mile program and allows Ugandan researchers at MUST to independently secure NIH grants (Ugandan members of our collaboration have been awarded R01 grants) and other awards. This effort hasn’t been without its struggles, with MGO’s growth marked by struggles with lack of adequate resources leading to the need for ongoing technical support from MGH. Capacity building grants historically provided through the NIH and other organizations like the Ewing Kauffman Foundation are critical opportunities to strengthen local administrative capacity, and the global research community must continue to advocate for increased Indirect Costs (IDCs) to international partners, which we feel would improve both research capacity and quality [30].

Additionally, partnerships should delineate a process and timeline for when health system strengthening projects would be led independently by local actors. Having explicit criteria and a goal timeline would help ensure transparency and accountability for all parties and allow for periodic evaluation of the project and its impact. As noted earlier, after a recent evaluation of our collaboration, our teams have developed a framework for evaluation that focuses on equity in funding and opportunity to help ensure we are meeting our shared goals. We as a team believe that through shared priorities with clear criteria and timelines, we can work towards improving the longevity, sustainability, and equity of health system strengthening projects.

The path to locally run health system strengthening projects is especially difficult due to funding imbalances between partners. In Uganda, there remains limited public funding and private philanthropy, which makes this transition especially difficult. Our partnership had several meetings on First Mile’s programmatic sustainability as the project neared completion. The collaboration’s support for the MGO has allowed for local investigators and clinicians to apply and win health system strengthening projects. Additionally, we found success in advocating that small aspects of First Mile, rather than the program as a whole, be taken over by the Ugandan Ministry of Health. While some aspects of the program are now locally owned, our collaboration has set new goals and timelines to continue this transition.

Secondly, our team feels strongly that research and clinical care must go hand in hand in global health work. In impoverished areas like Uganda, there are many competing healthcare priorities for the available funding. Before First Mile, the MGH-MUST-MRRH collaboration had been primarily focused on research. While research capacity at MUST and MRRH grew, clinical capacity did not significantly change, raising questions around the partnership’s work and which activities were prioritized [31]. Not only is improving clinical delivery in Uganda a moral imperative, but we have found that combining direct clinical work with ongoing research has improved the quality of both and strengthened the collaboration between our institutions. Ours is not the only example: our colleagues at the AMPATH Consortium, Partners in Health, and Médecins Sans Frontières have underscored the ethical obligation of treating patients and supplementing medical care with locally relevant research [32–34].

Finally, in looking to the future of First Mile and similar health projects, we consider how to make such programs more sustainable and how local providers can be empowered in leading the charge for health system strengthening. In terms of sustainability, we feel that constant engagement with governmental leaders is critical but insufficient. The Ugandan Ministry of Health works with a limited budget and a myriad of competing priorities. Given that improved health is multidisciplinary, we urge future groups to work with various government bodies (education, food and agriculture, etc.) and social organizations (NGOs, community organizers, advocacy groups). As an example, we partnered with a Ugandan civil society organization that specialized in community mobilization. Working with this group allowed us to improve access to services provided through First Mile for diverse communities in the southwestern region and to more effectively amplify our message in the country.

Our analysis is limited by this being a moderate-length health-system strengthening project in one region of Uganda. Given the myriad health activities co-occurring during the period of our work, including the COVID-19 pandemic, it is difficult to show clear impacts on regional health improvements, with most results being limited to process and outcome indicators. Additionally, our analysis is limited by the fact that all authors are members of the collaboration and leaders of the First Mile program, With this in mind, we commissioned a third-party evaluation of the MGH-MUST-MRRH collaboration with an eye towards equity in 2024, which has informed this work and our future goals as a collaborative. We believe such external evaluations to be critical for North-South partnerships as we continue to push for equity in global health.

## Conclusions

The First Mile program was ultimately successful in meeting the initial broad goals set in 2018, aiming to improve scholarship, clinical care, community-based medical training, and research and innovation in southwestern Uganda. Primary challenges we faced included sustainability of program activities, a need for centralized leadership, and frustrations around program decisions. Transparent decision-making and a Ugandan program director, built on a 20-year collaboration, aided in increasing transparency and helping with conflict resolution. We believe the First Mile project provides a strong example of how North-South partnerships can leverage health system strengthening grants using private philanthropy to respond to local needs. Our team learned a tremendous amount during this iterative process and hope that our struggles will make it easier for other teams to succeed in the future. We believe strongly that similar work in the future should develop local systems for sustainability (i.e. grants offices for financial management), engage with government early and often for program visibility, focus on both clinical and research work, and remain responsive to local needs as they arise and take advantage of synergies across disciplines and disease areas.

## Data Availability

The datasets used and/or analyzed are available from the corresponding author on reasonable request.
